# 3D Ultrasound in Pelvic Floor: Is It Useful as a Prognostic Tool in Type of Labor Development and Subsequent Pelvic Floor Diseases?

**DOI:** 10.3390/ijerph191811479

**Published:** 2022-09-13

**Authors:** Juan A. Barca, Coral Bravo, Santiago García Tizón, Rocío Aracil-Rodriguez, Juan Manuel Pina-Moreno, Ignacio Cueto-Hernández, Maria P. Pintado-Recarte, Melchor Alvarez-Mon, Miguel A. Ortega, Juan A. De León-Luis

**Affiliations:** 1 Department of Public and Maternal and Child Health, School of Medicine, Complutense University of Madrid, 28040 Madrid, Spain; 2 Health Research Institute Gregorio Marañón, 28009 Madrid, Spain; 3 Department of Obstetrics and Gynecology, University Hospital Gregorio Marañón, 28009 Madrid, Spain; 4 Ramón y Cajal Institute of Healthcare Research (IRYCIS), 28034 Madrid, Spain; 5 Department of Medicine and Medical Specialties, Faculty of Medicine and Health Sciences, University of Alcalá, 28801 Alcalá de Henares, Spain; 6 Immune System Diseases-Rheumatology, Oncology Service an Internal Medicine (CIBEREHD), University Hospital Príncipe de Asturias, 28801 Alcalá de Henares, Spain

**Keywords:** 3D ultrasound, pelvic floor disorders, perineal tear, obstetric vaginal tear, obstetric injuries, levator ani muscle

## Abstract

The objective of our study is to determine the thickness of the pubovisceral fasciculus of the levator ani muscle and the area of the genital hiatus by means of three-dimensional perineal ultrasound, in pregnant women in the 2nd trimester, and to analyze the related maternal, perinatal and postpartum clinical variables. Furthermore, to compare the results of our study with two similar series previously published. An observational, prospective study of pelvic floor ultrasound was carried out, performed at week 20, whose delivery was attended in the obstetrics service of the Hospital General Universitario Gregorio Marañón de Madrid (HGUGM), during the period of August from 2021 to June 2022. Maternal, ultrasound, perinatal and postpartum clinical variables were collected from each participant. During the study period, a total of 54 patients were included in it. The mean gestational age at which the ultrasound was performed was 19.81 ± 0.91 weeks. In relation to the ultrasound variables, the mean thickness of the pubovisceral muscle was 0.87 ± 0.13 cm (95% CI, 0.64–1.38 cm), while, in the plane of minimum dimension of the genital hiatus, the hiatal area at rest was 13.41 ± 3.22 (95% CI, 4.60–18.78) cm^2^. There is a significant correlation between the age of pregnant women (over 35 years of age) and the increase in the area of the genital hiatus (r = 0.295, *p* = 0.031). 3D ultrasound of the pelvic floor performed at week 20 of gestation can to be an effective, non-invasive, reproducible and cheap tool in the prognosis of the development of labor and of possible subsequent perineal dysfunctions.

## 1. Introduction

The relationship between the Levator ani muscle (LAM) and pelvic floor morbidity has been studied in different studies. In fact, De Lancey et al. have related LAM trauma or increased urogenital hiatus with loss of pelvic floor support [1,2]. Pelvic floor ultrasound can increasingly offer us a prognostic window of how the pelvic structures are, to relate them to the different pathologies of the pelvic floor. For their part, Dietz and Hoyte demonstrated, through the use of 3D ultrasound of the pelvic floor and MRI, that there was a relationship between LAM defects and pelvic organ prolapse (POP), producing an increase in the urogenital hiatus, even suffering from a unilateral or bilateral avulsion of the muscle [3,4,5,6]. In fact, there are several exams including manual examination, ultrasound examination, ultrasound elastography exam as well combined ultrasound and biochemical exams (collagen and elastin) to evaluate the soft tissue of pelvic floor.

During childbirth, the LAM plays a fundamental role since it is the soft tissue that defines the biomechanical properties of the birth canal [7]. In fact, in other studies, the biomechanical properties of LAM in childbirth are very important, having a direct relationship between distention capacity and the type of delivery and even a shorter second stage labor [8,9].

LAM avulsion and consequently an increase in the genital hiatus are related to different pelvic floor dysfunctions (PFD), such as pelvic organ prolapse (POP) [10,11,12,13], urinary incontinence [14,15,16,17], fecal incontinence [18,19,20] and sexual dysfunctions [7,21,22].

Pelvic floor ultrasound has an increasing clinical application, for the measurement and visualization of different structures, such as the LAM, urogenital hiatus, LAM avulsions, etc. and its performance shows cheap, simple and very precise results, compared to MRI, in the visualization of the different perineal structures [23,24,25,26,27,28,29,30,31,32].

Transperineal ultrasound is considered the gold standard for exploration through imaging of the pelvic floor, being a tool for detecting different dysfunctions of the anterior, central or posterior compartment of the female genital sphere [33].

The LAM can elongate in very different ways in patients as a result of biomechanical stress or perineal trauma during childbirth, as well as having different morphological characteristics, among different patients that facilitate or slow down the second labor in childbirth [17], being able to generate in turn, damage to the different perineal structures, which can lead postpartum and even years later, to different dysfunctions of the pelvic floor, such as urinary incontinence (UI), pelvic organ prolapse (POP), anal incontinence (AI) or sexual dysfunctions, because of the likelihood of soft tissue trauma.

The objective of our study is to determine the thickness of the pubovisceral fasciculus of the LAM and the area of the genital hiatus by means of three-dimensional perineal ultrasound in 2nd trimester pregnant women and to analyze the related maternal, perinatal and postpartum clinical variables. Furthermore, to compare the results of our study with two similar series previously published.

## 2. Materials and Methods

A descriptive, observational and cross-sectional study has been carried out on a sample of pregnant women who attended the consultation for the routine ultrasound of week 20 at the Hospital General Universitario Gregorio Marañón, during the period between August and October 2021.

Among the inclusion criteria were a pregnant woman over 18 years of age, a singleton pregnancy without serious gestational pathology, a visit to perform a morphological ultrasound at week 20, and acceptance of informed consent. Exclusion criteria were delivery in another center and fetal death during pregnancy. Patients who did not respond to the postpartum questionnaire were excluded from the comparative analysis.

During the ultrasound examination, in addition to the obstetric ultrasound, measurements of the thickness of the pubovisceral fasciculus of the LAM and the area of the genital hiatus were made by three-dimensional transperineal ultrasound.

**Figure 1:**
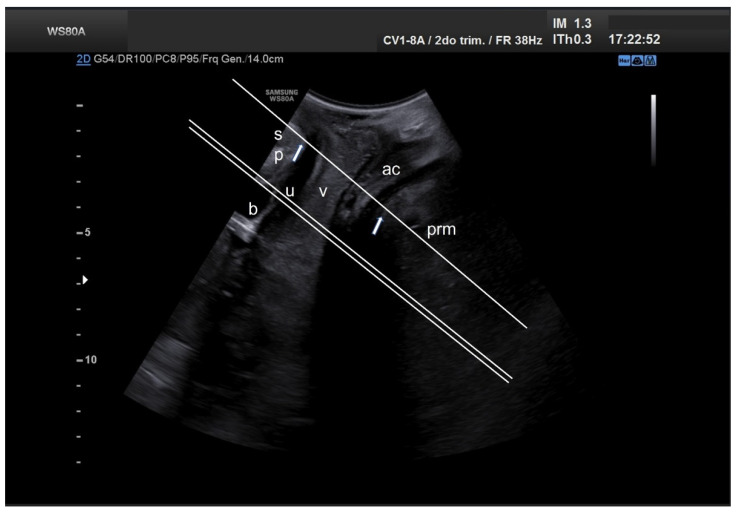
Mid-sagittal translabial two-dimensional pelvic floor ultrasound, showing the location of planes used for determining hiatal diameters and areas (single line) as well as pubovisceral muscle thickness and area (double line). ac, anal canal; b, bladder; prm, puborectalis muscle; sp, pubic symphysis; u, urethra; v, vagina.

**Figure 2:**
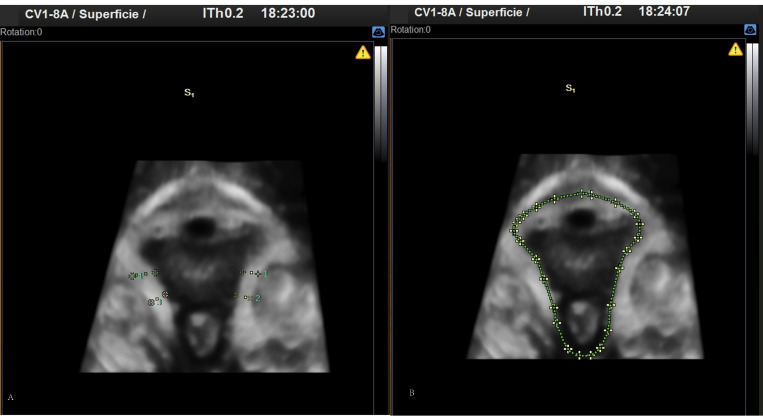
3D images obtained by perineal ultrasound. (**A**) Pubovisceral muscle thickness. (**B**) Perineal hiatus area.

Images were obtained by 3D translabial ultrasound in the supine position, using a SAMSUNG ultrasound machine, model WS80A, with a 3D-4D volumetric probe (4–7 MHz). The method used to obtain the anatomical dimensions of the thickness of the pubovisceral muscle and the genital hiatus is that described in the study published by Dietz et al. [34] after being considered reproducible in other studies [35,36,37]. In order to follow the methodology of the aforementioned studies and in relation to the anatomical structures used in these studies, the same cut-off points have been used for the genital hiatus area and the thickness of the pubovisceral muscle, so that it can be reproducible and comparable in the 3D study of the pelvic floor structure. The ultrasound probe was oriented in the midsagittal plane. The acquisition angle was set at the transducer maximum of 70°. The volumetric images were obtained at rest and with an empty bladder, after checking in the midsagittal plane in 2D. Figure 1 shows the location of the plane taken in 2D, to determine the diameter of the hiatus and the thickness of the pubovisceral fasciculus. The plane of minimum dimensions of the hiatus was identified in the midsagittal plane, as the minimum distance between the hyperechoic posterior aspect of the pubic symphysis and the hyperechoic anterior border of the levator ani muscle, just after the anorectal image. When a correct 2D ultrasound image was obtained, the 3D volume was automatically obtained using the system’s 3D function, which maximizes acquisition quality and is saved in system memory. On this image, 4 measurements of the thickness of the pubovisceral fasciculus were made, from the internal muscle edge to the external In to In method on both sides at positions 3 and 5 o’clock, calculating the mean between the measurements to obtain the average thickness of the pubovisceral fasciculus of each patient. In addition, the perineal hiatus area was measured on the same image by delimiting its contours (Figure 2).

**Figure 3:**
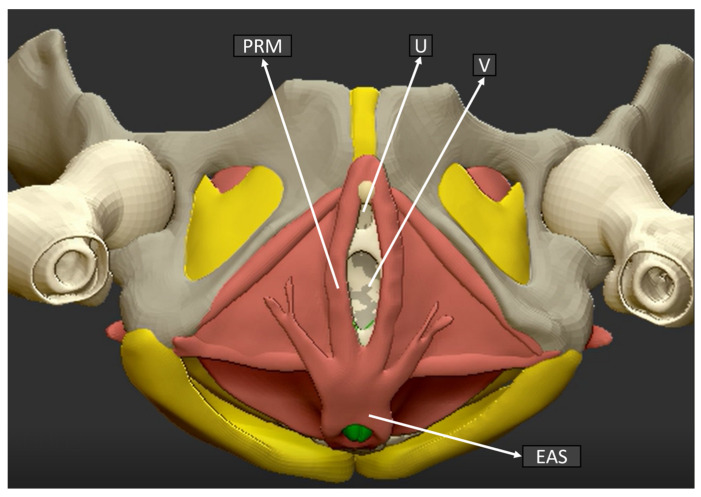
Pelvic floor anatomical structure coronal plane. (U) Urethra, (V) Vagina, (PRM) Puborectalis muscle, (EAS) External anal sphincter.

In Figure 3, an anatomical sketch of the coronal plane of the pelvic floor is shown in order to illustrate the exact plane and structures obtained by the 3D ultrasound examination.

In addition to the ultrasound examination, patients were offered to participate in a postpartum health questionnaire that assessed symptoms of perineal dysfunction. Those who accepted received a copy of the questionnaire to be completed so that they knew the questions beforehand. A non-standardized questionnaire was defined (Annex 1that included questions related to the possibility of suffering from some perineal dysfunction. Pelvic floor ultrasound measurement was performed by a single clinician during the study.

The surveys were conducted by telephone call three months after delivery, in the period between April and June 2022.

Maternal, ultrasound, perinatal and postpartum clinical variables were collected for each participant and included in a database created for this purpose.

### Statistic Analysis

The data obtained from the study were included in a Microsoft Office Excel database, version 2019 (Microsoft, Redmond, WA, USA) and the statistical analysis was performed with SPSS version 25 programs (IBM Corp. Released 2017. IBM SPSS Statistics for Windows, Version 25.0. Armonk, NY, USA) and Epidat 3.1 program for epidemiological data analysis. Version 3.1, Conselleria de Sanidade, Xunta de Galicia, Santiago, Spain; Pan American Health Organization (PAHO-WHO); CES University, Colombia. For each group, the descriptive parameters mean, standard deviation and 95% confidence interval were calculated for all the quantitative variables, and the percentage relative frequencies were calculated for the qualitative variables. A comparative analysis of the clinical variables of the patients was carried out based on the two groups of patients established according to presenting values of pubovisceral and hiatal thickness or equal to or less than the mean and above the mean. To calculate the effect correlation of each variable, a univariate logistic regression analysis was carried out to determine the value of the OR.

Subsequently, of the most representative variables in the results of the comparison of the groups, a calculation of the correlation between these ultrasound measurements and the most representative variables in the results of the comparison of the two groups was carried out.

To compare differences in anatomical measurements, statistical differences were calculated from the summary data (Mean, SD, and sample size) using Student’s *t*-test. A *p* < 0.05 has been defined for statistical significance.

The study protocol received the approval of the Ethics Committee for Medical Research of our center (Code: MSP-1, 13 July 2021).

## 3. Results

During the study period, 54 patients from our center who met the inclusion criteria were recruited. Maternal and ultrasound variables could be collected in all recruited patients.

No cases of LAM injury were detected in the ultrasounds performed, not even in patients with previous deliveries.

Nine (16.67%) patients were excluded due to delivery in another center and 1 (1.85%) patient due to fetal death, therefore 44 patients were included for the study of perinatal and postpartum variables.

Table 1 describes the maternal clinical and ultrasound variables. The mean age of the patients was 33.80 ± 4.59 years and they attended on average at 19.81 ± 0.91 weeks of gestation. It stands out that 44.4% of the patients were older than 35 years, as well as a multiparity percentage of 44.4%.

In relation to the ultrasound variables, the mean thickness of the pubovisceral muscle was 0.87 ± 0.13 cm (95% CI, 0.64–1.38 cm), while, in the plane of minimum dimension of the genital hiatus, the hiatal area at rest was 13.41 ± 3.22 (95% CI, 4.60–18.78) cm^2^.

55.8% of deliveries were induced, with a frequency of 4.5% instrumental delivery and 9.1% cesarean section. 43.2% of perineal tears have been described in vaginal deliveries.

In order to analyze the results in the different variables, the patients have been classified into two groups based on the measurement of the pubovisceral muscle, classifying them into those with a measurement equal to or less than the mean (0.87 cm) and above the mean. In addition, they have been classified into two groups in relation to the size of the genital hiatus, also taking the mean of its area as the cut-off point (equal to or less than the mean of 13.41 cm^2^ and greater than the mean).

Table 1 describes the results of the clinical, delivery and postpartum variables in the two groups with the highest risk of possible association with pathology, which are the group with thickness of the pubovisceral muscle equal to or less than the average and the group with area of the pubovisceral muscle. higher than average hiatus.

Table 2 shows the analysis of the association of the different most relevant clinical variables with risk groups. There is no significant association between age over 35 years and the thickness of the pubovisceral fasciculus (r = 0.008, *p* = 0.952), however, this association has been established with the increase in the genital hiatus (r = 0.295, *p* = 0.031). None of the other variables studied have shown statistical significance, including parity, labor induction, or the presence of IU or POP postpartum. Only the frequency of labor induction is close to statistical significance, although its clinical etiological relationship is not clear.

In addition, it describes the degree of association through odds ratio. The variables with the greatest difference in cases, in the comparison between groups in Table 1 and that may have greater clinical relevance with the thickness of the pubovisceral muscle, are maternal age (OR = 1.03; *p* = 0.51), labor induction (OR = 3.34; *p* = 0.060) and urinary incontinence (OR = 1.97; *p* = 0.336). While the main variables with greater relevance to the genital hiatus area are maternal age (OR = 3.38; *p* = 0.033), multiparity (OR = 2.10; *p* = 0.183), urinary incontinence (OR = 1.33; *p* = 0.666) and pelvic organ prolapse (OR = 2.25; *p* = 0.261).

As previously mentioned, a comparison of the measurements obtained in our study with those published in the works of Dietz et al. and Yang et al. has been incorporated into the analysis. in non-pregnant women. The results of this comparison are shown in Table 3.

It can be noted that, in our study on pregnant women, the pubovisceral muscle is thicker than in the aforementioned studies (*p* < 0.001). However, these differences do exist in the area of the genital hiatus, which is higher in our study (*p* = 0.002). The age of the patients also differs between these studies, being higher in our sample, which could justify this second point.

## 4. Discussion

In this study we have performed a three-dimensional ultrasound evaluation of pregnant women in the second trimester of pregnancy in order to determine the measurements of the thickness of the LAM pubovisceral fascicle and the area of the genital hiatus.

The mean area of the genital hiatus appears to be related to age, being higher in the group of women older than 35 years (OR = 3.38; *p* = 0.033). It is widely described in other studies that age has an impact on the increase in different PFDs [37,38,39,40], so we believe that it is important to take this variable into account in pregnant women when it comes to anticipate the possibility of the appearance of any perineal dysfunction in the postpartum period. Multiple studies have linked increased genital hiatus area to POP [1,3,4,41,42]. The relationship shown in this study between age and the increase in the hiatal area corroborates these results and allows selecting those patients who present a high risk of suffering from UI or POP in the postpartum period.

POP occurs when there is a defect in the support mechanism of the different levels of suspension that Delancey et al. already described, in order to even be able to support normal intra-abdominal pressure [43,44]. In fact, Delancey et al. and other studies show that when the levator ani is damaged or weak, as can occur in an obstetric procedure, the area of the genital hiatus tends to increase [1,3,11,12,42]. In our study, we have not found any patient with unilateral or bilateral levator lesion, so we can think that this increase in the genital hiatus could be caused by the intra-abdominal pressure itself in relation to the fetus, as well as a possible weakness of the levator muscle. In non-pregnant women, POP occurs when the pelvic floor structures have compromised competence and/or integrity and must withstand sustained intra-abdominal pressure [3,7,17,44], so as we have observed in the results of the study, the pressure of the uterus itself during pregnancy and especially after week 20, on the entire suspension structure of the pelvic floor, can generate this increase in the area of the genital hiatus, and subsequently result in some type of POP. Although not statistically significant, in our study the risk of postpartum POP is doubled in pregnant women with an increase in the area of the genital hiatus at week 20 (OR = 2.25; *p* = 0.261).

In addition, we have been able to determine that in our study the measurement of the thickness of the pubovisceral muscle and the genital hiatus does not seem to be related to age or other clinical parameters such as parity. The only aspect related to thickness, in a significant way, has turned out to be the probability of labor induction, however, we do not know the underlying pathophysiological relationship if it exists. We have also not been able to find a significant association between variables related to childbirth and muscle thickness or hiatal area, despite the fact that they seem to be biologically related, such as the total duration of labor, the frequency of eutocic or instrumental vaginal delivery, or the of soft canal tears. This may be due to the low power due to the end of the study or to the fact that the examination during pregnancy around week 20 does not allow an association to be established with different events that may take place during childbirth.

In the evaluation of the risk factors for UI and POP, the presence of a lower-than-average thickness in the pubovisceral muscle shows an OR of 1.97 for postpartum IU, although without reaching statistical significance. In the case of increased genital hiatus, the OR for POP is 2.25, although with a *p* of 0.26. A higher *n* is likely to be needed to assess these outcomes, which are known risk factors for pelvic floor pathology. We must also take into account that the development of these pathologies can present years of latency, so that a single evaluation at 3 months postpartum may not adequately represent the evolution in the more distant future.

If we analyze our results by comparing them with the studies by Dietz et al. and Yang et al. the patients in our study present a larger area of the genital hiatus. This can be explained either by her gestational state in which the weight of the gravid uterus on this muscular diaphragm increases its diameter and is in turn related to the older average age of our population.

Another relevant aspect in the results of the study is the relationship that may exist between the decrease in thickness of the pubovisceral muscle and the performance of labor induction. In fact, labour induction has been studied as one of the important risk factors for pelvic floor tears [45,46,47,48]. In our study, patients who had a thickness of the pubovisceral fasciculus less than the average, have a higher probability of labour induction (OR = 3.34; CI 95% 0.94–11.85; *p* = 0.06). Therefore, an early detection of a thinner pubovisceral muscle (we can associate it with less muscle tone and strength), may indicate a higher risk of a perineal tear during childbirth, especially if it is an induced, long or instrumental delivery and its subsequent appearance of postpartum anal incontinence, due to the existing relationship already described in other studies [49,50,51,52,53,54].

Ultrasound is currently the fundamental tool in the study of the pelvic floor muscles, given its accessibility and low cost compared to other radiological techniques [55,56].

The possibility of obtaining these images has been very high, being able to capture volumes in all the patients recruited, so in practice it is a technique that is easy to acquire, minimally invasive and of reasonable cost compared to MRI. In addition, its performance during pregnancy can be framed within the obstetric ultrasound study and contribute to detecting and informing those patients with risk factors for pelvic floor pathology in the future and designing both primary prevention strategies (pelvic floor exercises, modify delivery care) as secondary if there are postpartum symptoms.

It is necessary to carry out more studies in pregnant patients, with more numerous samples that improve the statistical power and thus be able to determine which are the main risk factors associated with pelvic floor dysfunction, especially those antepartum that can be influenced in primary prevention. Interobserver studies are also advisable in order to compare the agreement between different sonographers. Despite other methods to assess the biomechanical parameters of the pelvic floor muscles have been described, such a biochemical markers like elastin and collagen, ultrasound assessment seems to be the first line of study given its availability, innocuity (no histology is required) and reproducibility [57,58].

A future research line could include the performance of a combination of imaging and biochemical studies in second or third trimester in order to obtain a more complete information of the status of the pelvic floor musculature and its possible behaviour under the stress of childbirth.

The timing of the 20-week examination was considered the optimal time for preventive action, in the case of high risk and as part of a complete examination that includes fetal morphological ultrasound, cervical measurement and second trimester analysis, although we can consider it a limitation of the study, not to have been able to perform measurements in the 32nd weeks to check for more significant associations. In fact, it would be interesting to be able to measure, biochemical parameters in the study population and combine them with the ultrasound study, to complete a pelvic floor examination.

## 5. Conclusions

3D ultrasound of the pelvic floor performed at week 20 of gestation can be an effective, non-invasive, reproducible and cheap tool in the prognosis of the development of labor and possible subsequent perineal dysfunction.

## Figures and Tables

**Table 1:** Characteristics of the study population. *N* values differ inside groups because of missing data of patients without childbirth variables and postpartum variables (*N* = 10).

		Overall *N* = 54		Pubovisceral Muscle Thickness ≤ 8.71 (cm) *N* = 30		Genital Hiatal Area ≥ 13.41 (cm^2^) *N* = 26	
		*n* (%)	SD	*n* (%)	SD	*n* (%)	SD
Maternal variables	Maternal age at birth > 35 (Years)	24 (44.4)		14 (46.7)		16 (61.5)	
	BMI	24.1	±5.32	24.72	±6.11	24.93	±6.16
	Caucasic ethnic	44 (81,50)		26 (86.7)		22 (84.6)	
	Multiparity	24 (44.4)		13 (43.3)		14 (53.8)	
	Gestational age at ultrasound (weeks)	19.81	±0.91	19.97	±1.03	19.88	±0.99
Ultrasound variables	Cephalic circunferenre (mm)	174,59	±11.25	175.47	±10.78	176.85	±9.83
	Estimated fetal weight (gr)	349,46	±74.28	359.53	±80.95	372.19	±83.82
	Genital hiatal area (cm^2^)	13.41	±3.22	13.74	±3.06	N/A	N/A
	Pubovisceral muscle thickness (cm)	0.87	±0.13	N/A	N/A	0.86	±0.12
Chilbirth variables	Gestational age at delivery (weeks)	38.93	±1.74	38.69	±2.02	38.38	±2.06
	Induction delivery	24 (55.8)		17 (68.0)		11 (55.0)	
	Delivery time (Hours)	7.97	±5.36	7.56	±5.26	7.49	±5.44
	Instrumental delivery	2 (4.5)		0 (0.0)		1 (4.8)	
	Caesarean section	4 (9.1)		2 (7.7)		0 (0.0)	
	Episiotomy procedure	14 (31.8)		8 (30.8)		7 (33.3)	
	Perineal tear at delivery	19 (43.2)		2 (7.7)		8 (38.1)	
	Weight of newborn (gr)	3212.67	±421.66	3164.81	±449.91	3288.75	±514.19
Postpartum variables	Urinary Incontinence	13 (30.2)		9 (36.0)		7 (33.3)	
	Anal incontinence	3 (7.0)		0 (0.0)		1 (4.8)	
	Pelvic organ prolapse	11 (25.6)		4 (16.0)		7 (33.3)	
	Dyspareunia	9 (20.9)		4 (16.0)		3 (14.3)	

**Table 2:** Correlation between variables representative of the study population and the cutoff measure for pubovisceral muscle thickness and genital hiatus area.

	Pubovisceral Muscle Thickness ≤ 8.71 (mm)				Genital Hiatal Area ≥ 13.41 (cm^2^)			
		95% CI				95% CI		
	Odds Ratio	Inferior	Superior	*p*	Odds Ratio	Inferior	Superior	*p*
Maternal age at birth > 35 (Years)	1.03	0.35	3.04	0.951	3.38	1.10	10.35	0.033
Multiparity	0.90	0.31	2.66	0.854	2.10	0.71	6.26	0.183
Induction delivery	3.34	0.94	11.85	0.06	0.94	0.28	3.14	0.920
Urinary Incontinence	1.97	0.50	7.82	0.336	1.33	0.36	4.92	0.666
Pelvic organ prolapse	0.30	0.07	1.25	0.098	2.25	0.55	9.25	0.261

**Table 3:** Patient sonographic and demographic data of the pelvic floor in tow series with different stage. Data are presented as mean ± SD (range). Comparison between these groups from the summary of data (mean, SD and sample size). * *p* value < 0.001 comparison with Yang et al. [35] study. ** *p* value < 0.001 comparison with Dietz et al. [34] study. *** *p* value < 0.001 comparison with Dietz et al. [34] study.

Variable	Dietz et al. [34]	Yang et al. [35]	Present Study	*p*
Sample	52 non pregnant women	48 non pregnant women	54 pregnant women	
Age (Years)	20.4 ± 1.49 (18–24)	26.6 ± 4.70 (19–38)	33.8 ± 4.59 (22–43)	<0.001
Body mass index (kg/m^2^)	23.5 ± 3.63 (18.8–33.6)	20.1 ± 2.10 (16.10–23.80)	24.10 ± 5.32 (17.30–44.37)	0.501 *
Pubovisceral muscle thickness (cm)	0.73 ± 0.16	0.84 ± 0.17 (0.48–1.22)	0.87 ± 0.13 (0.84–0.91)	0.316 **
Genital hiatal area (cm^2^)	11.25 ± 2.70 (6.34–18.06)	11.69 ± 2.18 (5.68–16.38)	13.41 ± 3.22 (12.53–14.29)	0.002 ***

## Data Availability

The data from this study are available at the obstetrics service of the Hospital General Universitario Gregorio Marañón in Madrid and will be provided upon request.

## Abbreviation

OR	Odds Ratio
C-Section	Cesarean birth
CC	Cephalic circumference
EFW	Estimated fetal weight
UI	Urinary incontinence
FI	Fecal incontinence
AI	Anal incontinence
POP	Pelvic organ prolapse
CI	Confidence interval
SD	standard deviation
BMI	Body mass index
LAM	Levator ani muscle
PFD	Pelvic floor diseases
HGUGM	Hospital General Universitario Gregorio Marañón de Madrid
